# Supplementary material for: Detecting haplotype-specific transcript variation in long reads with FLAIR2
Source: bioRxiv. 2023 Jun 12:2023.06.09.544396. Preprint. [Version 1] doi: 10.1101/2023.06.09.544396 (PMC10312636; doi:10.1101/2023.06.09.544396)
Supplement: Supplement 5 [file NIHPP2023.06.09.544396v1-Supplement-5.pdf]

SFig 1

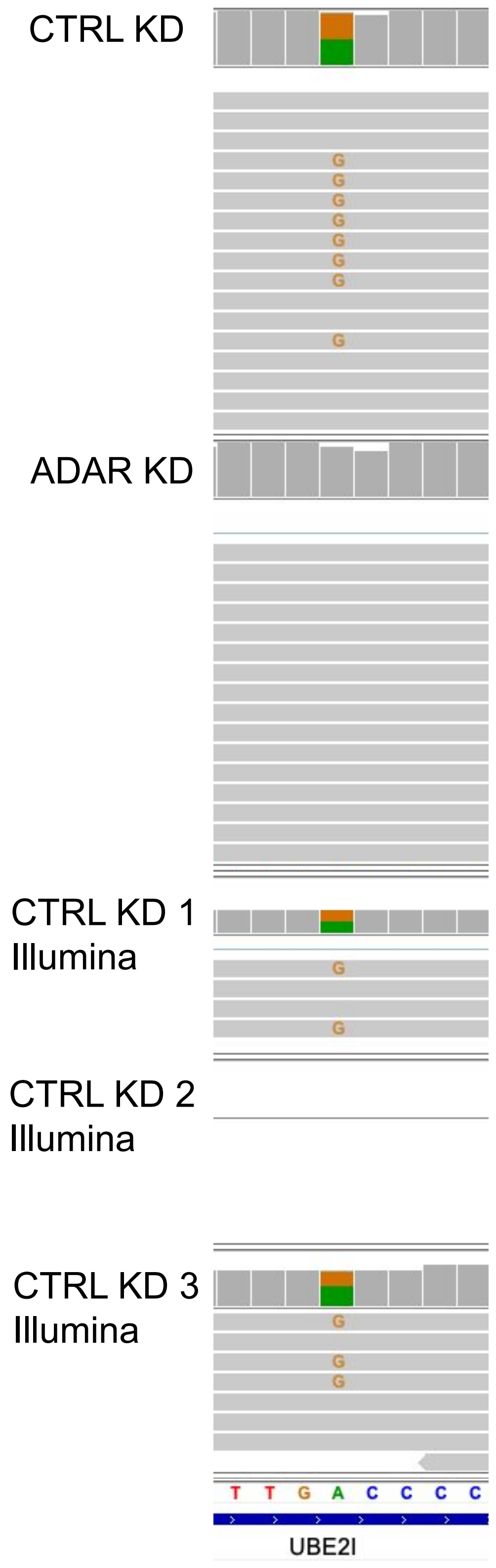

Supplemental Figure 1 | Example of novel A-to-I editing found with nanopore data. IGV shots of nanopore and Illumina data aligned to hg38. There were no reads aligning to *UBE2I* in the second Illumina CTRL KD replicate.

Supplementary Table 2

Gene Set Name	p-value	FDR q-value
DNA metabolic process	2.75E-13	4.27E-09
DNA repair	2.21E-11	1.72E-07
Cellular response to DNA damage stimulus	1.80E-10	9.34E-07
Cellular response to stress	3.39E-10	1.32E-06
Chromosome	1.45E-09	4.49E-06
Cell cycle	3.27E-09	8.47E-06
Transferase complex	7.38E-09	1.62E-05
Catalytic complex	8.34E-09	1.62E-05
Small molecule metabolic process	6.89E-08	1.19E-04
Cellular macromolecule biosynthetic process	1.72E-07	2.67E-04

bioRxiv preprint doi: https://doi.org/10.1101/2023.06.09.544396; this version posted June 12, 2023. The copyright holder for this preprint (which was not certified by peer review) is the author/funder, who has granted bioRxiv a license to display the preprint in perpetuity. It is made available under aCC-BY 4.0 International license.

Supplementary Table 2 | GO terms associated with haplotype-specific transcripts found in hybrid castaneus x 129 mouse data.
